# *PsRPs26*, a 40S Ribosomal Protein Subunit, Regulates the Growth and Pathogenicity of *Puccinia striiformis* f. sp. *Tritici*

**DOI:** 10.3389/fmicb.2019.00968

**Published:** 2019-05-10

**Authors:** Bing Wang, Na Song, Chunlei Tang, Jinbiao Ma, Ning Wang, Yanfei Sun, Zhensheng Kang

**Affiliations:** ^1^State Key Laboratory of Crop Stress Biology for Arid Areas, College of Plant Protection, Northwest A&F University, Yangling, China; ^2^Hunan Provincial Key Laboratory for Biology and Control of Plant Diseases and Insect Pests, College of Plant Protection, Hunan Agricultural University, Changsha, China

**Keywords:** ribosomal subunit, growth, *Puccinia striiformis*, wheat, RPs26

## Abstract

Eukaryotic ribosomes are essential for proliferation, differentiation, and cell growth. RPs26 is a ribosomal subunit structural protein involved in the growth and development process. Little is known about the function of *PsRPs26* in pathogenic fungi. In this study, we isolated the RPs26 gene, *PsRPs26*, from *Puccinia striiformis* f. sp. *tritici* (*Pst*). *PsRPs26* contains a eukaryotic-specific Y62–K70 motif and is more than 90% identical with its ortholog gene in other fungi. PsRPs26 was found to be localized in both the nucleus and cytoplasm. Expression of *PsRPs26* increased when wheat seedlings were inoculated with the *Pst* CYR31 isolate. Moreover, knockdown of *PsRPs26* by a host-induced gene silencing system inhibited growth and limited urediospore production in *Pst*. Our discovery that *PsRPs26* may contribute to the pathogenicity of *Pst* and open a new way in the pathogenic function of *PsRPs26* in cereal rust fungi.

## Introduction

Ribosomes are responsible for protein synthesis and essential to many organisms, ranging from bacteria to animals (Karsi et al., 2002). Ribosomal proteins (RPs) and ribosomal RNA (rRNA) are macromolecular components of the ribosome (Ferreira-Cerca et al., 2007). Mammalian ribosomes consist of 79 RPs and four rRNA species (Wool et al., 1995; Warner and Nierras, 1998). The eukaryotic 80S ribosome is composed of a small (40S) and a large subunit (60S). The 40S ribosome contains the 18S rRNA and 32 RPs, whereas the 60S ribosome is composed of three rRNAs and 47 RPs (Wool, 1979; Wool et al., 1995). As ribosomes exist in a wide spectrum of organisms, RPs have been highly conserved – 35 RP homologs exist in eubacteria, archaea, and eukarya (Verschoor et al., 1998; Belyy et al., 2016).

RPs26 is a structural protein of the ribosomal 40S subunit. In 1977, the first RPs26 was identified in rat livers (Collatz et al., 1977). Subsequently, the corresponding gene was isolated from human cDNAs (Filipenko et al., 1998). RPs26 was also cloned from the giant Panda (Hou et al., 2010). *RPs26a* and *RPs26b* are found to be 97% identical in the yeast *Saccharomyces cerevisiae* (Strittmatter et al., 2006). There is no significant eubacterial counterpart of *RPs26*; but S18 is the functional homolog of RPs26 in eubacteria, which contains a similar rRNA-contacting structural motif (Malygin and Karpova, 2009). In humans, the connection between mRNA and Rps26 was established via the Y62–K70 motif (62-YXXPKXYXK-70) conserved in eukaryotes (Sharifulin et al., 2011). Recently, RPs have been proposed as the model for understanding post-transcriptional regulation of gene expression.

It has been demonstrated that ribosome biogenesis is essential for cell growth and development. A genetic study found that depleting Sfp1, which controls the expression of numerous genes related to ribosome assembly, resulted in reduced cell size (Jorgensen et al., 2002). RPs26 is also involved in multiple growth and development processes. RPs26 genes in Diamond-Blackfan anemia patients affect the function of the proteins in rRNA processing (Doherty et al., 2010). Rps26 suppressed the splicing of its own pre-mRNA when it was expressed in *Escherichia coli*, suggesting some feedback mechanism might be controlling Rps26 synthesis (Malygin et al., 2003; Ivanov et al., 2005). In *S. cerevisiae*, Rps26B can support the growth of single yeast cells but not filamentous growth. Rps26Ap appears to be a product of the yeast translation machinery, as it is not only required for general translation but also functions in the diploid pseudohyphal growth and regulation of haploid adhesive in yeast (Strittmatter et al., 2006; Belyy et al., 2016). Additionally, Rps26 is shown to participate in endoplasmic reticulum stress in yeast (Steffen et al., 2012).

Wheat (*Triticum aestivum* L.) is an important food crop worldwide, the grain qualities and production of which are greatly impacted by pathogens. Wheat stripe rust, caused by the fungus pathogen *Puccinia striiformis* f. sp. *tritici* (*Pst*), has become the largest biotic suppression factor to the yield of wheat, causing more than 90% production losses in a field (Wang et al., 2018). Additionally, *Pst* rapidly evolves new virulent rust fungal isolates to adapt to most race-specific host resistance genes (Fisher et al., 2012). *Pst* was found to complete its sexual stage in barberry (*Berberis shensiana*), leading to pathogenic variation in *Pst* (Zheng et al., 2013).

To study the epidemiology, biology, and pathogenic factors of *Pst*, a complementary DNA (cDNA) library was built from wheat seedlings inoculated with *Pst* CYR31 isolate (Ma et al., 2009). We identified a 26S ribosomal protein gene, *PsRPs26*, from the library. *PsRPs26* contains the eukaryotic-specific YxxPKxYxK motif. Functional characterization showed that knockdown of *PsRPs26* leads to suppressed fungal growth and development and limited urediospore production of *Pst.*

## Materials and Methods

### Plant Material and Treatments

*Puccinia striiformis* f. sp. *tritici* CYR31 isolate and wheat cultivar Suwon 11 (Su11) were used. Su11 is susceptible to *Pst* CYR31 isolate. Fresh urediniospores of the CYR31 isolate were collected from inoculated wheat leaves. The culture, inoculation, and incubation of Su11 followed the report by Kang et al. (2002). Inoculated and control wheat leaves were harvested at 24, 48, 120, 168, and 192 h post-inoculation (hpi), and inoculated leaves of barberry at 12 days post-inoculation (dpi) were collected. The plant samples were quickly frozen and stored at -80°C.

### Isolation of RNA and Quantitative Real-Time (qRT) PCR Analysis

For total RNA extraction, wheat leaves that were inoculated with *Pst* CYR31 isolate were extracted using TRIzol^TM^ reagent (Invitrogen, Carlsbad, CA, United States) following the recommended protocol. First-strand cDNA was synthesized using the qRT-PCR System (Promega Corp., Madison, WI, United States). qRT-PCR primer design and reaction conditions were based on Wang et al. (2009). The *Pst* elongation factor *PsEF* was selected as the internal reference gene for the qRT-PCR analyses. The specific primers of *PsRPs26* used in the qRT-PCR analysis were listed in Supplementary Table S1. All relative gene expression results were assayed using the comparative 2^-ΔΔCT^ method (Livak and Schmittgen, 2001).

### Sequence Analysis, Alignment, and Structure Prediction

The homologs are in a set of fungal genomes deposited in the National Center for Biotechnology Information (NCBI^1^) databases and Ensembl Fungi^2^. Phylogenetic trees were constructed with the neighbor-joining algorithm MEGA5 (Tamura et al., 2011). To identify intraspecific polymorphisms, we compared the coding regions of 11 *Pst* isolates CYR32, CYR23, CYR31, CYR33, V26, Su11-4, Yr9, PST-21, PST-78, PST-08/21, and PST-87/7. To identify nucleotide substitutions in *PsRPs26*, we performed PCR amplifications with the cDNA of these isolates (Wang et al., 2017). Multiple protein sequence alignments were generated using CLUSTALW (Thompson et al., 1994).

### Subcellular Localization

A *pCAMBIA-1302-PsRPs26-GFP* fusion vector was used to verify the subcellular localization. We performed a transient expression analysis using *Nicotiana benthamiana* to examine the subcellular localization of PsRPs26. The reconstructed vector *pCAMBIA-1302-PsRPs26-GFP* and empty vector were transformed into strain GV3101 of *Agrobacterium tumefaciens* by electroporation. The leaves of 4-week-old *N. benthamiana* were transiently transformed with strains carrying the empty vector or *PsRPs26-GFP*. Green fluorescent protein (GFP) signals were examined with an Olympus BX-51 microscope (Olympus Corp., Tokyo).

### *PsRPs26* Gene Silencing Using Host-Induced Gene Silencing (HIGS)

Barley stripe mosaic virus (BSMV) γRNA-based vectors used to knockdown the expression of *PsRPs26* were followed as described by Holzberg et al. (2002). The sequenced PCR product of *PsRPs26* were digested with *Not*I and *Pac*I and inserted into the digested BSMV:g vector. The entire second leaf was infected with virus transcripts by mildly rubbing it on the leaf surface at the two-leaf stage (Hein et al., 2005). The wheat phytoene desaturase (*TaPDS*) was used as a control. Three independent sets of wheat plants were used for each experiment (BSMV:*TaPDS*, BSMV:γ, and BSMV: *PsRPs26*). The fourth leaves were inoculated with fresh urediniospores of the *Pst* virulence race CYR31 10 days later. The disease phenotypes were recorded and photographed at 14 days post-inoculation (dpi). The fourth leaves with *Pst* were excised at 24, 48, and 120 hpi for RNA isolation and histological determination.

### Histological Determination of Fungal Growth

Wheat samples were stained as described previously by Wang et al. (2007). Transparent leaf segments were examined using an Olympus BX-51 microscope for haustorial mother cells, hyphal lengths, and infection areas. Thirty to fifty infection sites were examined for each treatment in each biological replication. Hyphal lengths, infection areas, and haustorial mother cells were calculated using DP-BSW software. Statistical analyses were performed with SPSS software.

## Results

### Identification of a 26S Ribosomal Protein Gene From *Puccinia striiformis*

**FIGURE 1:**
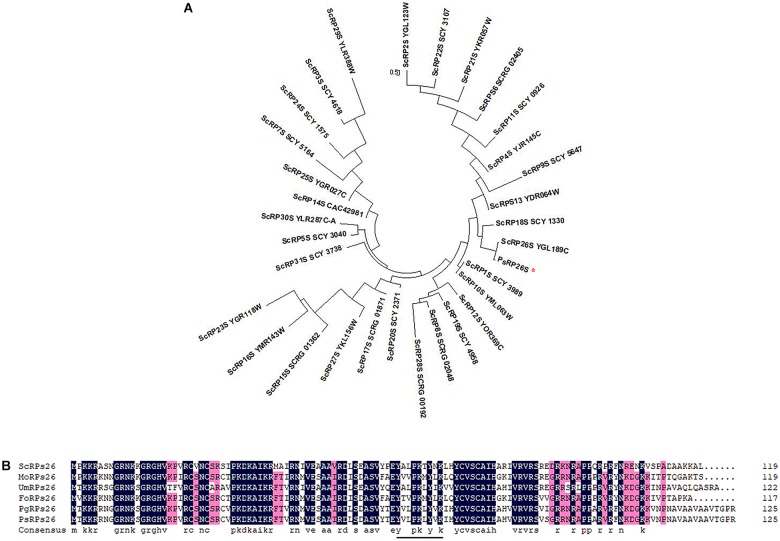
Phylogenetic analysis and multiple sequence alignment of PsRPs26. **(A)** Phylogenic analysis of PsRPs26 and *Saccharomyces cerevisiae* RPs family members using MEGA 5.0 software. **(B)** Protein multiple alignment. Identical and similar amino acid residues are shaded in black and light gray, respectively. Ps, *Puccinia striiformis* f. sp. *tritici*; Pg, *Puccinia graminis* f. sp. *tritici*; Us, *Ustilago maydis*; Mo, *Magnaporthe oryzae*; Fo, *Fusarium oxysporum*; and Sc, *Saccharomyces cerevisiae*.

One transcript that encodes a putative 40S ribosomal protein subunit was identified by mapping the *Pst* genome (Zheng et al., 2013). The wheat cDNA sequence comprises of a 381-bp open reading frame (ORF) and encodes a protein of 126 aa. The corresponding protein was predicted to have a molecular weight of 13.95 kDa. To identify the subfamily of the 40S ribosomal protein subunits, a phylogenetic analysis was constructed using 31 different 40S ribosomal protein subunits of *S. cerevisiae* from Ensembl Fungi (see footnote 2). The results indicated that the deduced protein grouped with ScRP26S (Figure 1A). Additionally, a multi-sequence alignment was conducted with various RP26Ss obtained from Ensembl Fungi and NCBI. The predicted protein shares high similarity (99.21%) with PgRP26S in *Puccinia graminis* using BLASTP analysis. Further analysis confirmed its similarity to other fungal RPs26 proteins, including *Fusarium oxysporum* FoRPs26, *Ustilago maydis* UmRPs26, and *Magnaporthe oryzae* MoRPs26 (Figure 1B). Thus, this gene was designated as *PsRPs26*.

Most 40S ribosomal protein subunits are highly conserved. We compared the coding regions between 11 different *Pst* isolates. Compared with the *PsRPs26* sequence from CYR31, which is one of the predominant *Pst* isolates in China, only four synonymous substitutions were observed. However, no nucleotide substitutions in the YxxPKxYxK motif were found among the 11 *Pst* isolates (Supplementary Figure S1). These results indicated *PsRPs26* is highly conserved.

### PsRPs26 Localizes to the Nucleus and Cytoplasm

**FIGURE 2:**
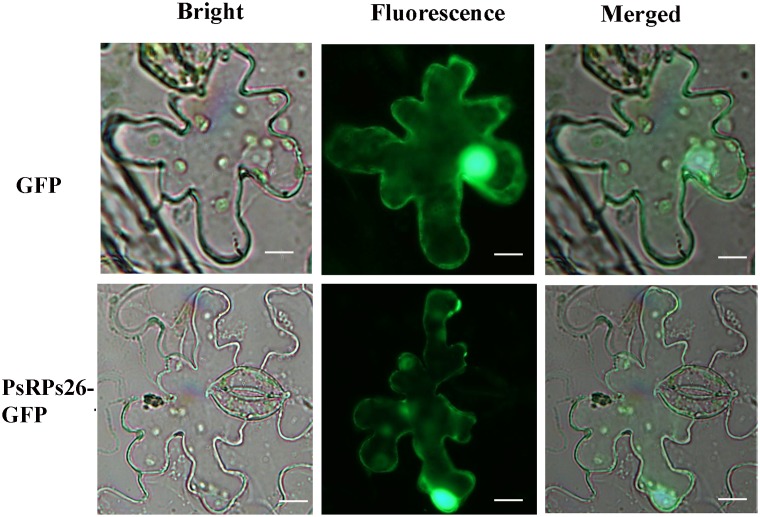
Subcellular localization of the PsRPs26 protein. The PsRPs26-GFP fusion protein and GFP (control) were transiently expressed in *Nicotiana benthamiana*. Bar = 20 μm. Similar results were obtained from three biological replicates.

A fusion *pCAMBIA-1302-PsRPs26-GFP* vector was constructed to determine the subcellular localization of PsRPs26. The control vector and *pCAMBIA-1302-PsRPs26-GFP* vector were transformed into *N. benthamiana* leaf cells. Microscopic observation showed that the PsRPs26-GFP fusion protein was located in the nucleus and cytoplasm (Figure 2). Florescent signals were localized in the nucleus, perinuclear area, and cytoplasm in the control. These results demonstrated that PsRPs26 is located in the cytoplasm and nucleus.

### *PsRPs26* Is Highly Induced During the Infection Stage of Wheat and Barberry

**FIGURE 3:**
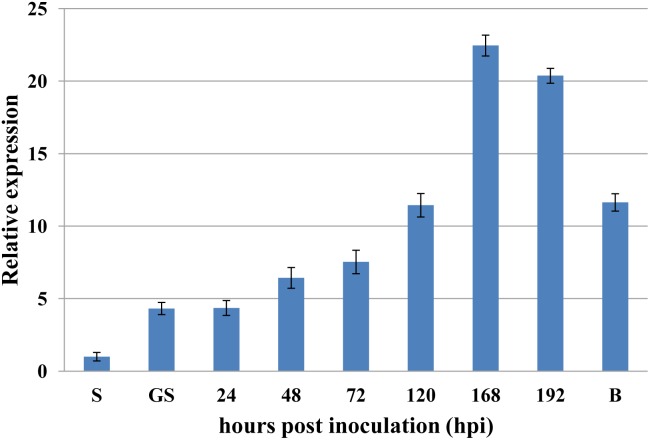
Expression of *PsRPs26* during the different infection stages of *P. striiformis.* The expression levels were normalized by *PsEF-1a*. S, urediniospores; GS, *in vitro* germ tube; B, infected *Berberis shensiana*. Mean expression values were calculated from three independent replicates.

To investigate if *PsRPs26* is involved in wheat and *Pst* interactions, qRT-PCR was performed to assay the transcript level of *PsRPs26* in different stages of *Pst*-host interactions. *PsRPs26* transcript levels increased as early as 24 hpi in *Pst*-wheat interactions. At 168 hpi with the CYR31 isolate, the transcript level of *PsRPs26* reached the maximum level of 22-fold compared with the control, which corresponds to the initiation of the sporulation stage. Furthermore, *PsRPs26* was highly expressed in *Pst*-barberry interaction (sexual reproduction, Figure 3). These results strongly supported our hypothesis that the transcript level of *PsRPs26* is induced during the *Pst* infection stage in host plants.

### Silencing of *PsRPs26* Attenuates *Pst* Growth and Development

A HIGS system with BSMV was used to further characterize the function of *PsRPs26* during *Pst* infection. At 10-day dpi with the virus, the BSMV:γ (control) and BSMV:*PsRPs26*-infected leaves displayed mild chlorotic mosaic symptoms. There was no other obvious defect on leaves infected virus. Furthermore, we used the recombinant virus BSMV:*TaPDS* to silence the wheat *PDS* gene, causing severe chlorophyll photo bleaching under the same conditions. These results proved that the HIGS system was functioning successfully.

To determine how *PsRPs26* participates in *Pst* growth and development, we observed the cytological changes in wheat seedlings with *PsRPs26* knocked down and infected with *Pst*. The fourth leaves of wheat infected with the virus were inoculated with *Pst* CYR31 isolate at 10 dpi. We then assayed for the haustoria and number of haustorial mother cells, as well as the hyphal length and the number of hyphal branches. In the BSMV:*PsRPs26*-treated leaves, the length of the infection hyphae and number of haustoria clearly decreased. But there were no significant differences in the number of hyphal branches and haustorial mother cells (Figure 5A,B). At 48 hpi with the CYR31 isolate, hyphal growth was significantly inhibited in the *PsRPs26*-silenced plants (Figure 5C). Compared to the control, *Pst* hyphal colony size was significantly suppressed (*P* < 0.05) in the BSMV:*PsRPs26*-treated wheat leaves at 120 hpi (Figure 5A,D). These results indicated that *PsRPs26* is involved in *Pst* growth and development during the infection stage in wheat.

### Transient Silencing of *PsRPs26* Significantly Limits Urediospore Production of *Pst*

**FIGURE 4:**
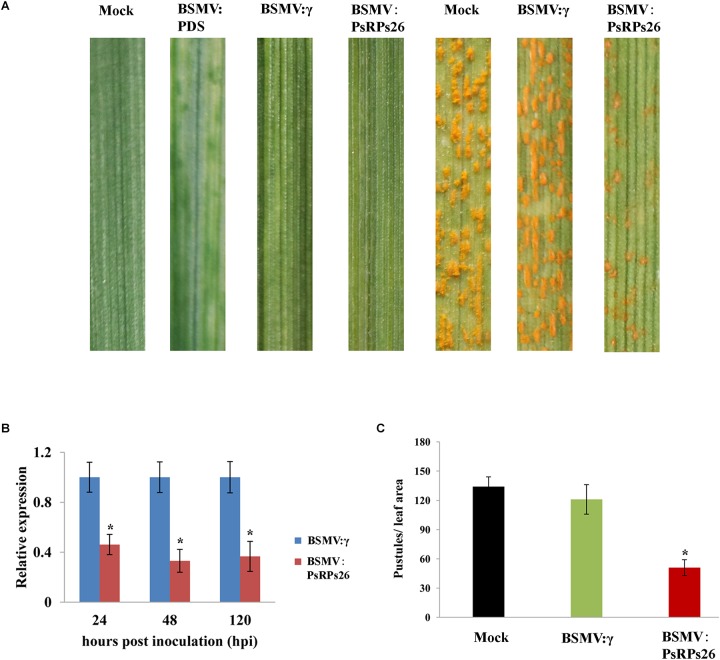
Functional characterization of *PsRPs26* during the interaction between wheat and stripe rust using host-induced gene silencing. **(A)** Phenotypic changes in the fourth leaves of plants pre-inoculated with the positive control vector (BSMV-PDS), FES buffer (CK), or empty BSMV vector (BSMV:γ). Phenotypes for the fourth leaves inoculated with the *P. striiformis* f. sp. *tritici* (*Pst*) CYR31 race at 14 dpi. **(B)** Relative transcript levels of *PsRPs26* in the silenced leaves. BSMV:γ leaves infected with the *Pst* CYR31 race were used as a control. **(C)** Quantification of uredinial density in *PsRPs26* knockdown plants 14 dpi with the CYR31 isolate. Means and standard deviations were calculated from three independent replicates. Significant differences were determined using Student’s *t*-test: ^∗^*P* < 0.05.

After 14 dpi, there were masses of urediospores on the CYR31-infected wild-type seedlings and BSMV:γ infection seedlings (control). Interestingly, leaves treated with BSMV:*PsRPs26* exhibited increased resistance to the CYR31 isolate, as indicated by the limited urediospore production compared with the controls (Figure 4A). We then confirmed the silencing efficiency of *PsRPs26* in the HIGS system using qRT-PCR. We found that the transcript levels of *PsRPs26* were reduced by 54, 67, and 63% at 24, 48, and 120 hpi, respectively (Figure 4B). The pustule density on the wheat leaves infected with BSMV:*PsRPs26* decreased by 58% (Figure 4C). These results suggested that the *PsRPs26* gene contributes to the pathogenicity of *Pst*.

**FIGURE 5:**
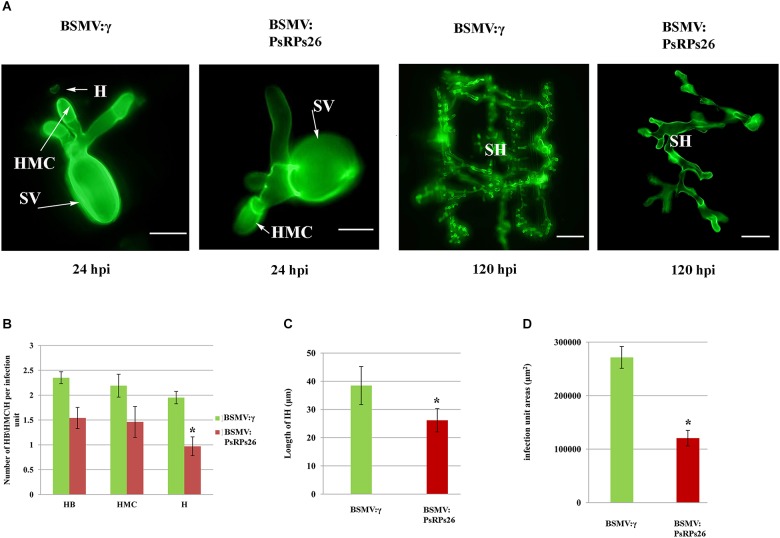
Histological determination of fungal growth in host cells. **(A)** Cytological observation of fungal development in *PsRPs26* knockdown wheat leaves inoculated with the *Pst* CYR31 race. Leaves were sampled at 24 and 120 hpi with the CYR31 isolate. **(B)** The number of haustoria was significantly reduced in the *PsRPs26*-silenced plants at 24 hpi with the CYR31 isolate. **(C)** Significant decrease in the length of the infection hyphae in the *PsRPs26*-silenced plants at 48 hpi with the CYR31 isolate. **(D)** *PsRPs26* -silenced plants show a significant decrease in infection unit area at 120 hpi. H, haustorium; SH, secondary hypha; HMC, haustorial mother cells; SV, substomatal vesicle (24 hpi, bar = 20 μm; 120 hpi, bar = 100 μm). Significant differences were determined using Student’s *t*-test: ^∗^*P* < 0.05.

## Discussion

Ribosome biogenesis is essential for proliferation, cell growth, differentiation, and development (Zhou et al., 2015). RPs26 is an elemental protein of the ribosomal 40S subunit. Since the discovery of the first RPs26 in rat livers, RPs26s have been identified in diverse species, ranging from fungi to animals (Collatz et al., 1977; Hou et al., 2010; Sharifulin et al., 2011). However, little is known about the pathogenic function of *RPs26* in cereal pathogenic fungi. In this study, we characterized the 40S ribosomal protein subunit, *PsRPs26*, from wheat leaves infected with *Pst*. *PsRPs26* contained the conserved Y62–K70 motif typical of the eukaryotic *RPs26* family. Interestingly, the *PsRPs26* motif is highly conserved across different *Pst* isolates. These results suggested that *PsRPs26* is one of the elemental structural proteins of the *Pst* ribosomal subunit.

Ribosomes are produced in the nucleus and are exported to the cytoplasm (Görlich and Kutay, 1999). Ribosomal subunits are organized within multiple cellular compartments: the cytoplasm, the nucleoplasm, and the nucleolus. 60S ribosomes undergo primary assembly in the cell nucleolus and are then transferred to the cytoplasm through the nucleoplasm for maturity (Nissan et al., 2002). Furthermore, RPS19 contributes to the function of ribosome biogenesis by translocating from the cytoplasm to the nucleus (Orrù et al., 2007). In this study, we determined that PsRPs26 localizes in both the nucleus and cytoplasm. These findings strongly support our hypothesis that PsRPs26 may function as a ribosomal protein subunit responsible for ribosome biogenesis.

Impairment of ribosome biogenesis can severely retard cell growth, as has been demonstrated in various eukaryotic systems. In *Drosophila*, *RPs* gene insufficiency reduces the number of ribosomes synthesized, leading to a small phenotype characterized by recessive lethality, delayed larval development, and small body size (Lambertsson, 1998; Saeboe-Larssen et al., 1998). RPL29-knockout mice displayed global skeletal growth defects that led to reduced postnatal viability in mammals (Kim-Safran et al., 2007). In *S. cerevisiae*, the RPS26A gene is essential for normal growth. Additionally, yeast cell is lethal when RPS26A and RPS26B genes are knocked out simultaneously (Strittmatter et al., 2006). In this study, we used the HIGS system to identify the function of *PsRPs26* to *Pst* growth and discovered that *PsRPs26* was knocked down during wheat-*Pst* interaction. *PsRPs26* was efficiently silenced by 50–60%. The formation and development of haustoria and haustorial mother cells were restricted during the early stage of *Pst* infection in plants with *PsRPs26* knocked down. Interestingly, *PsRPs26* was upregulated during *Pst*-barberry interaction. *Pst* infection was similar to that of wheat, including haustorium formation, and fungal colonization (Cheng et al., 2015). Furthermore, silencing of *PsRPs26* suppressed the formation of secondary hyphae and fungal colonization at 120 hpi with *Pst*, indicating that *PsRPs26* is involved in fungi development during infection.

*RPs26* function has been well studied in yeast and mammals. However, little is known regarding *PsRPs26* function in pathogenic fungi. In this study, the observed knock-down effect of *PsRPs26* during wheat-*Pst* interaction led to increased resistance against infection by the CYR31 isolate. However, there is little direct functional evidence to show which genes were involved in rust pathogenicity because mutagenesis and transformation protocols are lacking in *Pst*. There are two possible reasons that can explain how *PsRPs26* contribute to *Pst* pathogenicity. The first proposes that *PsRPs26* silencing suppresses *Pst* growth and development during infection, as indicated by the reduced number of uredinia on wheat leaves. Interestingly, *PsRPs26* was highly upregulated at 168–196 hpi with the CYR31 isolate, which corresponded with initiation of sporulation. *PsRPs26* was efficiently silenced, as characterized by limited urediospore production when compared to the controls. Thara et al. (2003) reported that a large number of fungal-like RPs, including *RPs26*, were characterized from susceptible wheat leaves infected with *Puccinia triticina* during sporulation, a stage likely to require vigorous protein synthesis. We, therefore, infer that the second reason may be silencing *PsRPs26* influences protein synthesis during sporulation. All these data suggested that *PsRPs26* contributes to *Pst* pathogenicity. However, the complex mechanism of how *PsRPs26* affects growth and pathogenicity needs to be studied further.

In conclusion, *PsRPs26* contained a eukaryotic-specific motif, suggesting that fungi and animals share conserved ribosomal protein components. PsRPs26 were localized in both the nucleus and cytoplasm, indicating that *PsRPs26* might participate in ribosome biogenesis. *PsRPs26* was induced in wheat infected with the *Pst* CYR31 isolate and contributed positively to the growth, development, and pathogenicity of *Pst*. Our findings may facilitate the design of fungicides for controlling disease in cereal crops.

## Author Contributions

BW carried out most of the experiments. BW, CT, and ZK wrote the manuscript. NS and JM performed the quantitative RT-PCR and analyzed the data. YS and NW grew the plant samples. NW collected all the phenotypic data and ZK revised the manuscript. All authors read and approved the final manuscript.

## Conflict of Interest Statement

The authors declare that the research was conducted in the absence of any commercial or financial relationships that could be construed as a potential conflict of interest.

## Footnotes

1 http://www.ncbi.nlm.nih.gov/

2 http://fungi.ensembl.org/

## Supplementary Material

FIGURE S1 Multiple sequence alignment of *PsRPs26* among different *Pst* isolates. The black line represents the Y62–K70 motif site.

TABLE S1 Primers used in this study.
