# Supplementary figures and images for: PsRPs26, a 40S Ribosomal Protein Subunit, Regulates the Growth and Pathogenicity of Puccinia striiformis f. sp. Tritici
Source: Front Microbiol. 2019 May 10;10:968. doi: 10.3389/fmicb.2019.00968 (PMC6523408; doi:10.3389/fmicb.2019.00968)

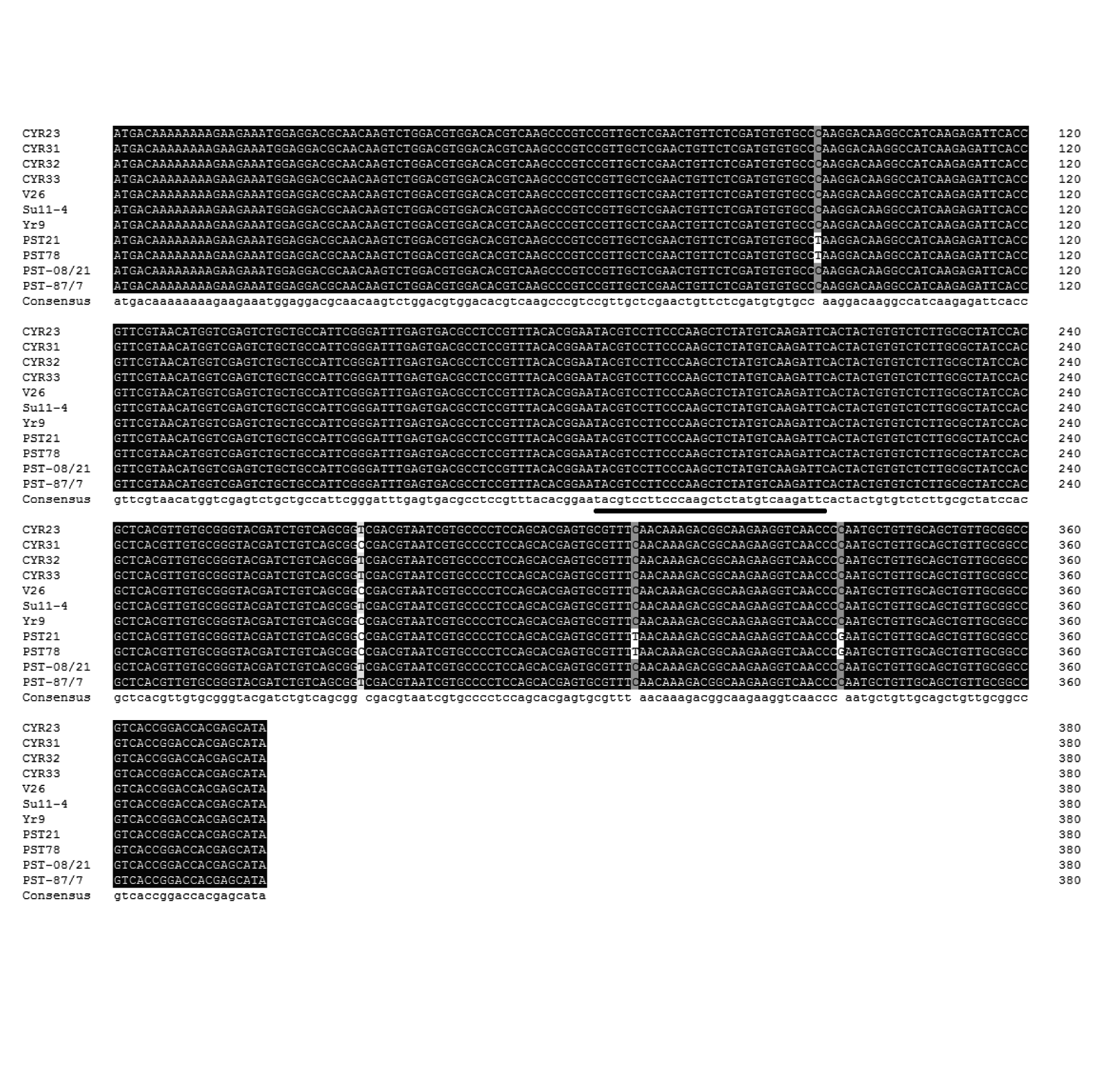

Supplement: FIGURE S1 — Multiple sequence alignment of PsRPs26 among different Pst isolates. The black line represents the Y62–K70 motif site. [file Image_1.TIF]
