# Supplementary material for: PsRPs26, a 40S Ribosomal Protein Subunit, Regulates the Growth and Pathogenicity of Puccinia striiformis f. sp. Tritici
Source: Front Microbiol. 2019 May 10;10:968. doi: 10.3389/fmicb.2019.00968 (PMC6523408; doi:10.3389/fmicb.2019.00968)
Supplement: TABLE S1 — Primers used in this study. [file Table_1.DOCX]

| Gene ID | Primers (5′-3′) |
| --- | --- |
| pCAMBIA-1302-PsRPs26F | CATGGTAGATCTGACTAGTATGGACCAGCACAACCAT |
| pCAMBIA-1302- PsRPs26R | GCCCTTGCTCACCATCCTAGGCTGCTGTAGGTGCAGCTG |
| VIGS- PsRPs26F | ATATTAATTAAGAAATGGAGGACGCAACAAGT |
| VIGS- PsRPs26R | TATGCGGCCGCATCCCGAATGGCAGCAGA |
| qRT-PCR- PsRPs26F | AACAAAGACGGCAAGAAGG |
| qRT-PCR- PsRPs26R | CCATAGTACAGCATCGGAGA |

**Table S1 Primers used in this study.**
